# OBATOCLAX and ABT-737 Induce ER Stress Responses in Human Melanoma Cells that Limit Induction of Apoptosis

**DOI:** 10.1371/journal.pone.0084073

**Published:** 2013-12-19

**Authors:** David Wroblewski, Chen Chen Jiang, Amanda Croft, Margaret L. Farrelly, Xu Dong Zhang, Peter Hersey

**Affiliations:** 1 School of Medicine and Public Health, University of Newcastle, New South Wales, Australia; 2 Kolling Institute, Royal North Shore Hospital, University of Sydney, New South Wales, Australia; Innsbruck Medical University, Austria

## Abstract

Anti-apoptotic Bcl-2 family proteins, in particular, Mcl-1, are known to play a critical role in resistance of human melanoma cells to induction of apoptosis by endoplasmic reticulum stress and other agents. The present study examined whether the BH3 mimetics, Obatoclax and ABT-737, which inhibit multiple anti-apoptotic Bcl-2 family proteins, would overcome resistance to apoptosis. We report that both agents induced a strong unfolded protein response (UPR) and that RNAi knockdown of UPR signalling proteins ATF6, IRE1α and XBP-1 inhibited Mcl-1 upregulation and increased sensitivity to the agents. These results demonstrate that inhibition of anti-apoptotic Bcl-2 proteins by Obatoclax and ABT-737 appears to elicit a protective feedback response in melanoma cells, by upregulation of Mcl-1 via induction of the UPR. We also report that Obatoclax, but not ABT-737, strongly induces autophagy, which appears to play a role in determining melanoma sensitivity to the agents.

## Introduction

Induction of apoptosis is an important mediator of cell death in response to a number of treatments such as chemotherapy and targeted therapies. Much is known about the regulation of apoptosis by the Bcl-2 family of proteins, in particular the complementary roles of pro-apoptotic BH3-only proteins and their opposing anti-apoptotic family members, which under normal conditions act in concert to regulate the induction of apoptosis. A shift in the balance towards pro-apoptotic Bcl-2 proteins sensitizes the cell towards induction of apoptosis, whereas relative dominance of the anti-apoptotic proteins provides resistance to apoptotic stimuli as is commonly found in treatment of malignancies [1].

Metastatic melanoma, in particular, has proven resistant to treatment with a variety of chemotherapeutic and biological agents [2]. This is believed to be largely due to activation of survival signalling pathways and upregulation of anti-apoptotic Bcl-2 family proteins [3,4]. Among the latter, the myeloid cell leukemia-1 (Mcl-1) protein plays a dominant role in resistance of melanoma to apoptosis. For instance, the sensitization of melanoma cells to TRAIL-induced apoptosis and by inhibition of the MEK signalling pathway was associated with downregulation of Mcl-1 [5-7]. Mcl-1 was also shown to be important for protection against apoptosis by Raf/MEK inhibitors [8]. Moreover, upregulation of Mcl-1 was critical for protection of melanoma cells against endoplasmic reticulum (ER) stress-induced apoptosis [7] and survival of melanoma cells treated with the proteasome inhibitor Bortezomib [9].

An attractive strategy to overcome the pro-survival effects of anti-apoptotic proteins is the use of so-called BH3 mimetics, which competitively bind to and inhibit anti-apoptotic Bcl-2 family proteins [10]. The most studied of these is the Abbott compound ABT-737, which was shown to be selective for Bcl-2, Bcl-XL and Bcl-W but not to Mcl-1 [11]. Subsequently, Mcl-1 was found to be a major cause of resistance of cancers to ABT-737 [12,13]. Several BH3 mimetics have however been reported to have a wider spectrum against anti-apoptotic proteins, including Mcl-1. These include the small molecule inhibitor TW-37, which was modelled on the binding of gossypol to pro-apoptotic Bim [14,15] and Obatoclax produced by Gemin-X. The latter was shown to have efficacy against non-small cell lung cancer, mantle cell lymphoma and multiple myeloma [16-18] and is in clinical trials in hematological malignancies[19,20]. In particular, Obatoclax was shown to overcome Mcl-1 mediated resistance to apoptosis [21,22]. Studies on human acute myeloid leukemia (AML) cells have also suggested that Obatoclax mediates the release of Bim and Bak from Mcl-1, resulting in release of cytochrome-c and apoptosis of AML cells [23].

In view of these results we have examined the effects of Obatoclax and ABT-737 in human melanoma cell lines and the mechanism of its effects. We report that the compounds induce apoptosis in melanoma cells, along with unexpected upregulation of a number of the Bcl-2 family proteins involved in regulation of apoptosis. We also identify activation of the unfolded protein response as a potential mechanism of resistance to BH3 mimetics in melanoma cells.

## Materials and Methods

### Ethics statement

This study complies with the Declaration of Helsinki and use of human melanoma tissue was approved by the Hunter New England Area Health Service Human Ethics Committee, approval number 05/02/09/3.02. Written consent was given by patients for the use of samples.

### Cell lines

Human melanoma cell lines Mel-RM, MM200, IgR3, Me1007, Me4405 and SKMel-28 have been described previously [5,24]. They were cultured in Dulbecco’s modified Eagle medium (DMEM) containing 5% fetal calf serum (FCS) (Commonwealth Serum Laboratories, Melbourne, Australia). Melanocytes were kindly provided by Dr. P. Parsons (Queensland Institute of Medical Research, Brisbane, Queensland, Australia) [25] and cultured in medium supplied by Clonetics. Isolation of melanoma cells from fresh surgical specimens was carried out as described previously [26]. The melanoma cells obtained after purification on Dynal beads were >95% melanoma, as defined by staining with an antibodies against CD45 and fibroblasts [26].

### Antibodies, recombinant proteins and other reagents

The rabbit polyclonal antibodies against phosphorylated eIF2α were from Stressgen (Victoria, British Columbia, Canada). The mouse mAb against Bcl-2 and Mcl-1, and the rabbit polyclonal Abs against Bcl-XL and GRP78, were purchased from Santa Cruz Biotechnology (Santa Cruz, CA). Mouse mAb against Noxa and the rabbit polyclonal Ab against Bim were from Imgenex (San Diego, CA). The rabbit polyclonal Ab against PUMA, LC3 and eIF2α, and the mouse Ab against p62, were from Cell Signalling Technology (Beverly, MA). The cell-permeable general caspase inhibitor Z-Val-Ala-Asp(OMe)-CH2F (z-VAD-fmk) was purchased by Calbiochem. Recombinant human TRAIL was supplied by Genentech Inc. (San Francisco, CA). The propidium iodide (PI), Bafilomycon A1 and the 1,2-bis(O-aminophenoxy)ethane-N,N,N,N-tetraacetic acid acetomethyl ester (BAPTA-AM) were purchased from Sigma Aldrich (Castle Hill, NSW, Australia). Fast SYBR® Green Master Mix was purchased from Applied Biosystems (Foster City, CA).

### Apoptosis

Quantitation of apoptotic cells by measurement of sub-G1 DNA content using the propidium iodide method was carried out as described elsewhere [27].

### Cell viability assay

The cytotoxic effect of compounds on melanoma cells was determined by use of VisionBlue Fluorescence Cell Viability Assay Kit (Biovision Inc., Mountain View, CA, USA) as described previously [28]. Briefly, cells were seeded at 5000 cells per well into flat-bottomed 96-well culture plates and allowed to grow for 16 hours prior to addition of compound in 100µl of fresh media. Following indicated treatment time, 10µl of VisionBlue reagent was added to each well, incubated for 4 hours and then read by the Synergy two multi-detection microplate reader (BioTek, Winooski, VT, USA) according to the manufacturer's instructions.

### Flow cytometry

Analysis of cells stained with propidium iodide or Calcium Green-1 was carried out using a Becton Dickinson (Mountain View, CA) FACSCanto flow cytometer.

### Western blot analysis

Western blot analysis was carried out as described previously [29]. Labelled bands were detected by Immun-Star horseradish peroxidase Chemiluminescent kit, and images were captured with the Bio-Rad VersaDoc image system (Bio-Rad, Regents Park, NSW, Australia). 

### XBP-1 mRNA splicing

The method of detecting the active spliced form of XBP-1 mRNA was described previously [6]. Briefly, RT-PCR products of XBP-1 mRNA were obtained from total RNA extracted using primers CGGTGCGCGGTGCGTAGTCTGGA (forward) and TGAGGGGCTGAGAGGTGCTTCT (reverse). Because a 26 bp fragment containing an Apa-L1 site is spliced upon activation of XBP-1 mRNA, the RT-PCR products were digested with Apa-L1 to distinguish the active (spliced) form from the inactive (unspliced) form. Subsequent agarose gel electrophoresis revealed the inactive form as two cleaved fragments and the active form as a non-cleaved fragment.

### Small interfering RNA

The small interfering RNA (siRNA) constructs used were obtained as the siGENOME SMARTpool reagents (Dharmacon, Lafayette, CO) and the siGENOME SMARTpool MCL-1 (M-004501-04-0010) and BCL-2 (M-003307-04-0010). The nontargeting siRNA control, siGENOME Non-Targeting siRNA Pool #1 (D-001206-13-20), was also obtained from Dharmacon. Transfection of siRNA pools was carried out as described previously [6].

### Real-time PCR

Real-time reverse transcription-PCR was performed using the 7900HT Fast PCR system (Applied Biosystems). 20µl mixture was used for reaction, which contains 4µl cDNA sample (0.5-1 µg/µl); 500nM forward primers for ATF6 (TCCACCTCCTTGTCAGCCCCT), IRE1α, (GCAAGCTGACGCCCACTCTGT) XBP-1 (GCACCTGAGCCCCGAGGAGA) or Mcl-1 (GGAAGGCGCTGGAGACCTTA) and 500 nM reverse primers for ATF6 (GCCCTGTTCCAGAGCACCCTGA), IRE1α (ACTTGACGTCCGTGCTGGGC), XBP-1 (TCATTCCCCTTGGCTTCCGCC) or Mcl-1 (CAACGATTTCACATCGTCTTCGT) and 10µl Fast SYBR® Green master mix. Analysis of cDNA for β-actin was included as a control. After incubation at 95°C for 20 seconds, the reaction was carried out for 40 cycles of the following: 95°C for 1 second and 60°C for 20 seconds. The threshold cycle value was normalized against β-actin cycle numbers. The relative abundance of mRNA expression of a control sample was arbitrarily designated as 1, and the values of the relative abundance of mRNA of other samples were calculated accordingly.

### Short hairpin RNA knockdown

Stable knockdown of individual genes was performed using Sigma MISSION Lentiviral Transduction Particles expressing a specific targeting set of short hairpin RNA (shRNA). Cells were seeded at 1 x 10^4^ per well in 96-well culture plates and transfected after 16 hours with individual clones from the Sigma MISSION shRNA targeting sets for ATF6 (SHCLNV-NM_007348), IRE1α (SHCLNV-NM_004133) and XBP-1 (SHCLNV-NM_005080) according to manufacturer’s instructions. Media was changed 24 hours following transfection, cells incubated overnight and then selected with 5 µg/ml puromycin until a stable population of resistant cells was achieved.

### Measurement of intracellular calcium levels

Cells were grown in medium with or without agents for indicated time points. Cells were collected, washed with PBS, and incubated with 1µM Calcium Green-1 (Molecular Probes, Eugene, OR) for 30 min before measurement of intracellular calcium levels by flow cytometry. Where indicated, cells were pretreated with 10µM BAPTA-AM 4 hours prior to addition of chemotherapeutic agents.

### Statistical analysis

Statistical significance was determined by use of Student’s t-test (parameters: two-tailed distribution, two-sample unequal variance). Where indicated, * P < 0.05, ** P <0.001.

## Results

### Melanoma cells are sensitive to Obatoclax but resistant to ABT-737

**Figure 1:**
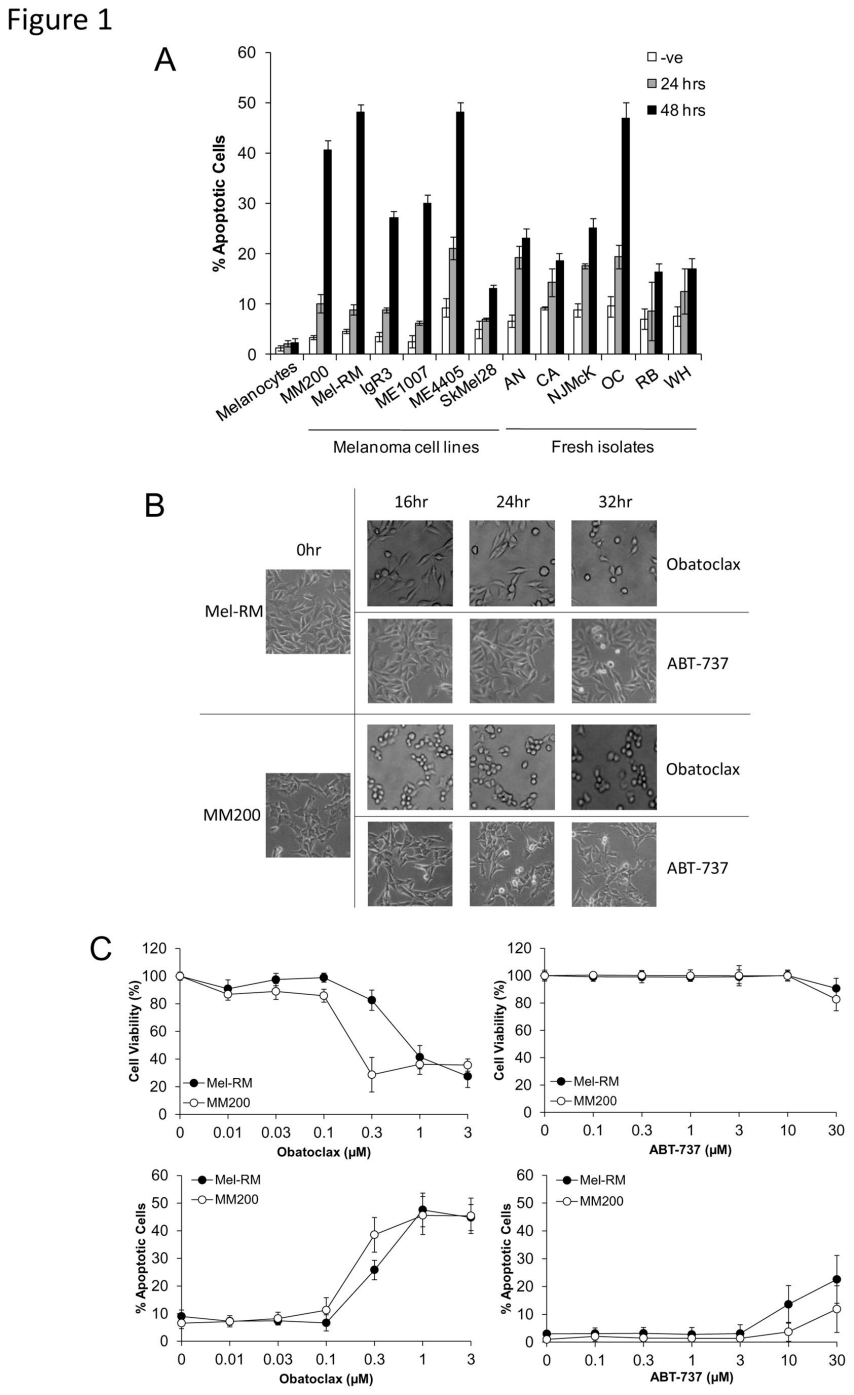
Obatoclax and ABT-737 induce apoptosis in melanoma cell lines. A. Summary of studies on a panel of melanoma cells and fresh melanoma isolates with normal cells (melanocytes). Following treatment of cells with Obatoclax (1μM) for 24 and 48 hours, cells were subjected to measurement of apoptosis by the propidium iodide method. *Points*, mean of three individual experiments; *bars*, SE. B. Treatment with Obatoclax, but not ABT-737, causes morphological changes in melanoma cells. Mel-RM and MM200 cells were treated with Obatoclax (1μM) and ABT-737 (10µM) for indicated periods. Photographs representative of cell population at each time point are displayed and are representative of three individual experiments. C. Dose titration of Obatoclax and ABT-737 in melanoma cells. Following treatment of Mel-RM and MM200 cells with various doses of Obatoclax or ABT-737 for 48 hours, cell viability was measured by MTS assay and apoptosis was measured by the propidium iodide method. *Points*, mean of three individual experiments; *bars*, SE.

Studies on a panel of human melanoma cell lines and fresh melanoma isolates indicated in Figure 1A, show that Obatoclax induced varying degrees of cell death, determined by measurement of subG1 DNA content, that was most evident in Mel-RM, MM200 and ME4405 cells, was maximal at 48 hours and was partially caspase dependent (Figure S1). Mel-RM and MM200 cells were chosen for subsequent studies as ME4405 does not express endogenous Bcl-2. As shown in Figure 1B, changes in cell morphology, including rounding up and shrinking, as well as an apparent decrease in the rate of cell proliferation, were observed as early as 16 hours following treatment with Obatoclax. Such morphological changes were not observed with ABT-737, which has affinity to all anti-apoptotic Bcl-2 proteins except for Mcl-1. Significant reduction of cell viability, as determined by MTS assay, was also detected from a concentration of 0.3µM of Obatoclax, however reduction of cell viability was not as pronounced following treatment with ABT-737. Further, apoptosis was detectable in Mel-RM and MM200 cells at a concentration of 0.3µM of Obatoclax and was maximal at 1µM. Sensitivity of melanoma cells to ABT-737 was comparatively low, with apoptosis detected only at doses of 10µM or higher, with Mel-RM more sensitive than MM200 (Figure 1C).

### Obatoclax and ABT-737 induce regulation of Bcl-2 family proteins with more marked upregulation of Mcl-1 by Obatoclax

**Figure 2:**
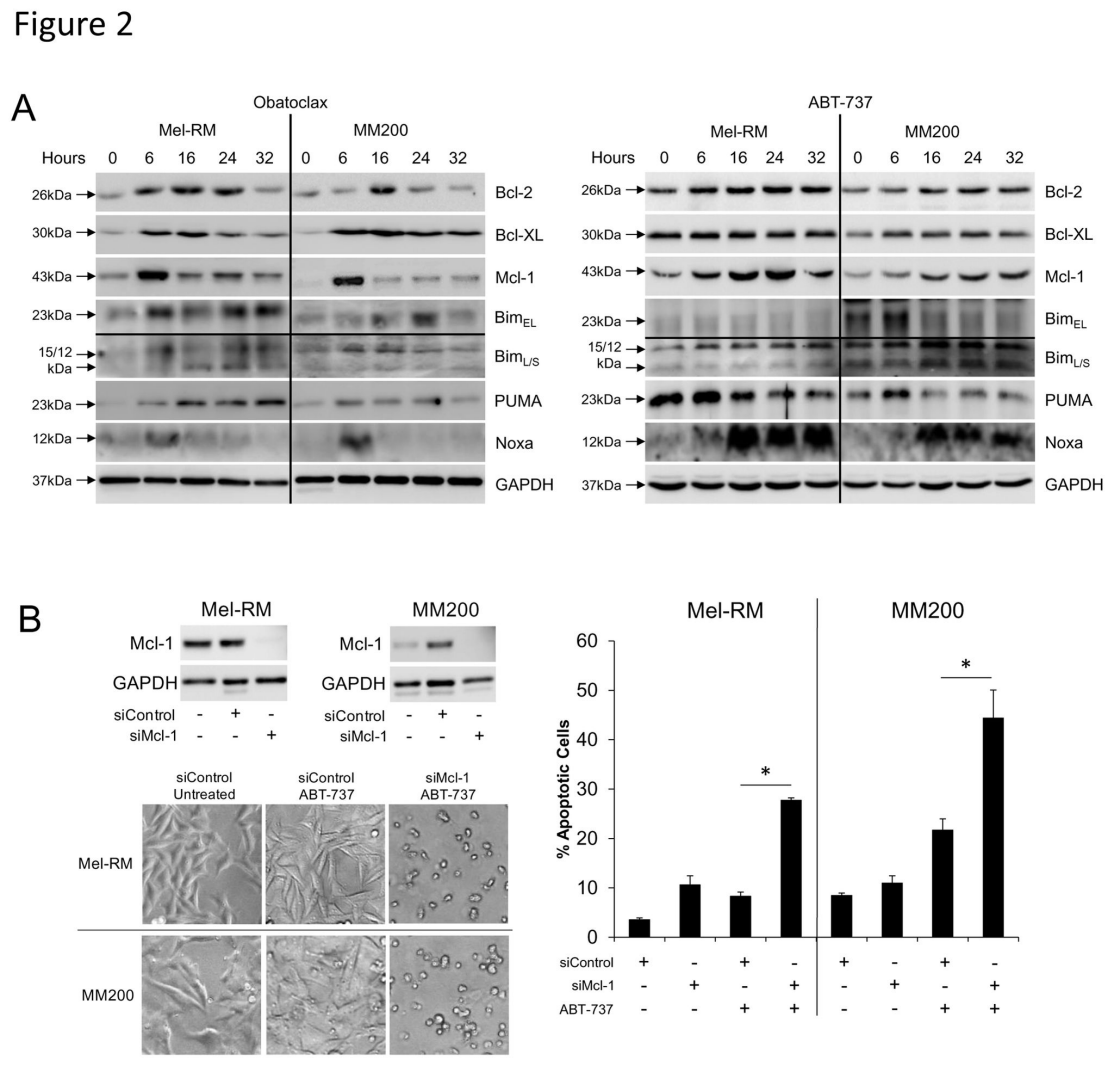
Obatoclax and ABT-737 induce regulation of Bcl-2 family proteins. A. Upregulation of BH3-only proteins and anti-apoptotic Bcl-2 family proteins occurs in response to Obatoclax. Whole cell lysates of Mel-RM and MM200 cells were subjected to Western blot analysis following treatment with Obatoclax (1µM) or ABT-737 (10µM) for indicated periods. Data are representative of three individual experiments. B. Knockdown of Mcl-1 increases cell sensitivity to ABT-737. Mel-RM and MM200 cells were transfected with either control or Mcl-1 siRNA before treatment with ABT-737 (10μM) for 24 hours. Cells were then subjected to measurement of apoptosis by the propidium iodide method. *Columns*, mean of three individual experiments; bars, SE. Knockdown of Mcl-1 was confirmed by Western blotting. Photographs representative of cell population at 24 hours are displayed and are representative of three individual experiments.

To better understand the mechanism of apoptosis induction we investigated the regulation of pro- and anti-apoptotic proteins by Obatoclax and ABT-737. As shown by the western blot studies in Figure 2A, induction of Bim occurred from 6 hours in Mel-RM and MM200 cell lines after exposure to each compound, while induction of PUMA was strongest at 16 hours in Mel-RM and 6 hours in MM200. Noxa was only transiently upregulated at 6 hours by Obatoclax in both cell lines, however significant and sustained upregulation occurred in response to ABT-737. An increase in Bcl-2 expression was maximal by 16 hours in both cell lines following treatment with both agents, in both cases decreasing to 32 hours thereafter. Obatoclax and ABT-737 caused strong upregulation of Bcl-XL in Mel-RM cells, decreasing after 16 hours, whereas upregulation of Bcl-XL by ABT-737 was slower and increased to 32 hours in MM200 cells. Obatoclax-induced Mcl-1 upregulation peaked transiently at 6 hours in both cell lines, while its expression in response to ABT-737 was sustained from 6 hours. Upregulation of Mcl-1 occurred transcriptionally as indicated by upregulation of Mcl-1 mRNA at 16 and 24 hours post-treatment (Figure S2).

As the melanoma cells were shown to be markedly more resistant to ABT-737 than Obatoclax, we used siRNA to knock down expression of Mcl-1, known to be the major difference between the specificity of both agents (Figure 2B). Predictably, sensitivity of Mel-RM and MM200 cells to ABT-737 was dramatically increased in cells lacking Mcl-1, with significant rounding of cells and greater detection of apoptosis as early as 24 hours. 

### Obatoclax and ABT-737 activate the unfolded protein response in melanoma cells

**Figure 3:**
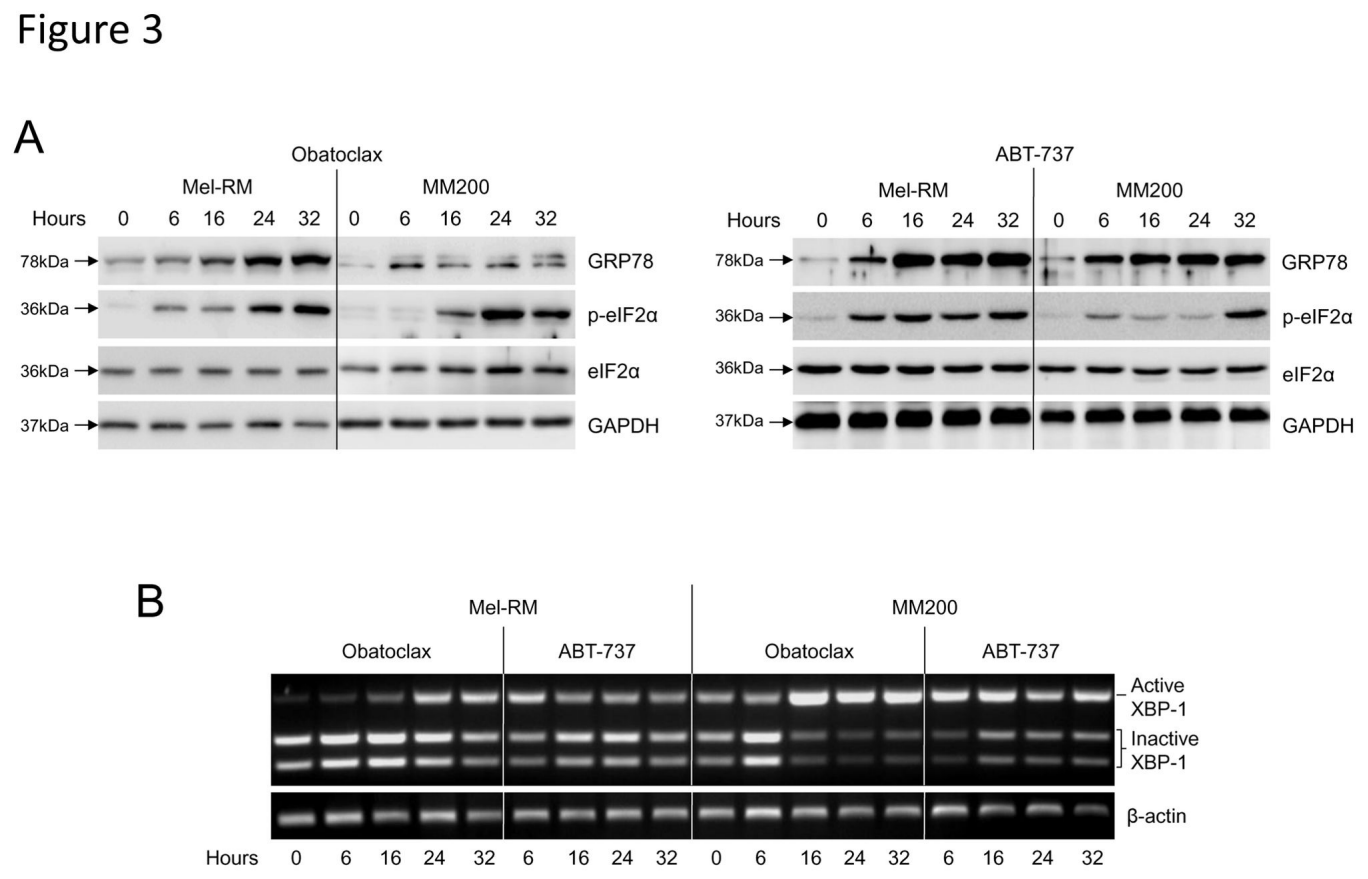
Obatoclax and ABT-737 activate the UPR in melanoma cells. A. Following treatment of melanoma cell lines Mel-RM and MM200 for indicated periods with Obatoclax (1nM) or ABT-737 (10µM), whole cell lysates were subjected to Western blot analysis for the ER stress markers GRP78 and phosphorylated eIF2α. Data are representative of three individual experiments. B. Expression of the active form of XBP-1 was determined by agarose gel electrophoresis following Apa-LI digestion of XBP-1 mRNA. Indicated is the longer (active) fragment and the two shorter (inactive) bands. Data are representative of three individual experiments.

We have previously shown that activation of the unfolded protein response (UPR) in melanoma cells can result in upregulation of Mcl-1 at the transcriptional level, a protective response to induction of ER stress [7]. We therefore investigated the ability of the agents to induce ER stress in melanoma cells as a potential mechanism for this protein regulation. Cells were treated with 1µM Obatoclax and 10µM ABT-737 for various times and activation of ER stress response pathways examined by Western blotting and semi-quantitative PCR. Glucose regulated protein 78 (GRP78) is a key marker of the ER stress response and was shown to be upregulated following treatment with both agents (Figure 3A). eIF2α in the PERK pathway was also phosphorylated after treatment. Additionally, an increase in the active spliced form of XBP-1, downstream of IRE1α, was detected from 6 hours of treatment with ABT-737 and 16 hours with Obatoclax (Figure 3B). Taken together, the activation of each ER stress marker in response to treatment indicated that both compounds were able to induce moderate levels of ER stress in melanoma cells.

### Inhibiting the UPR sensitises melanoma cells to Obatoclax- and ABT-737-induced cell death

**Figure 4:**
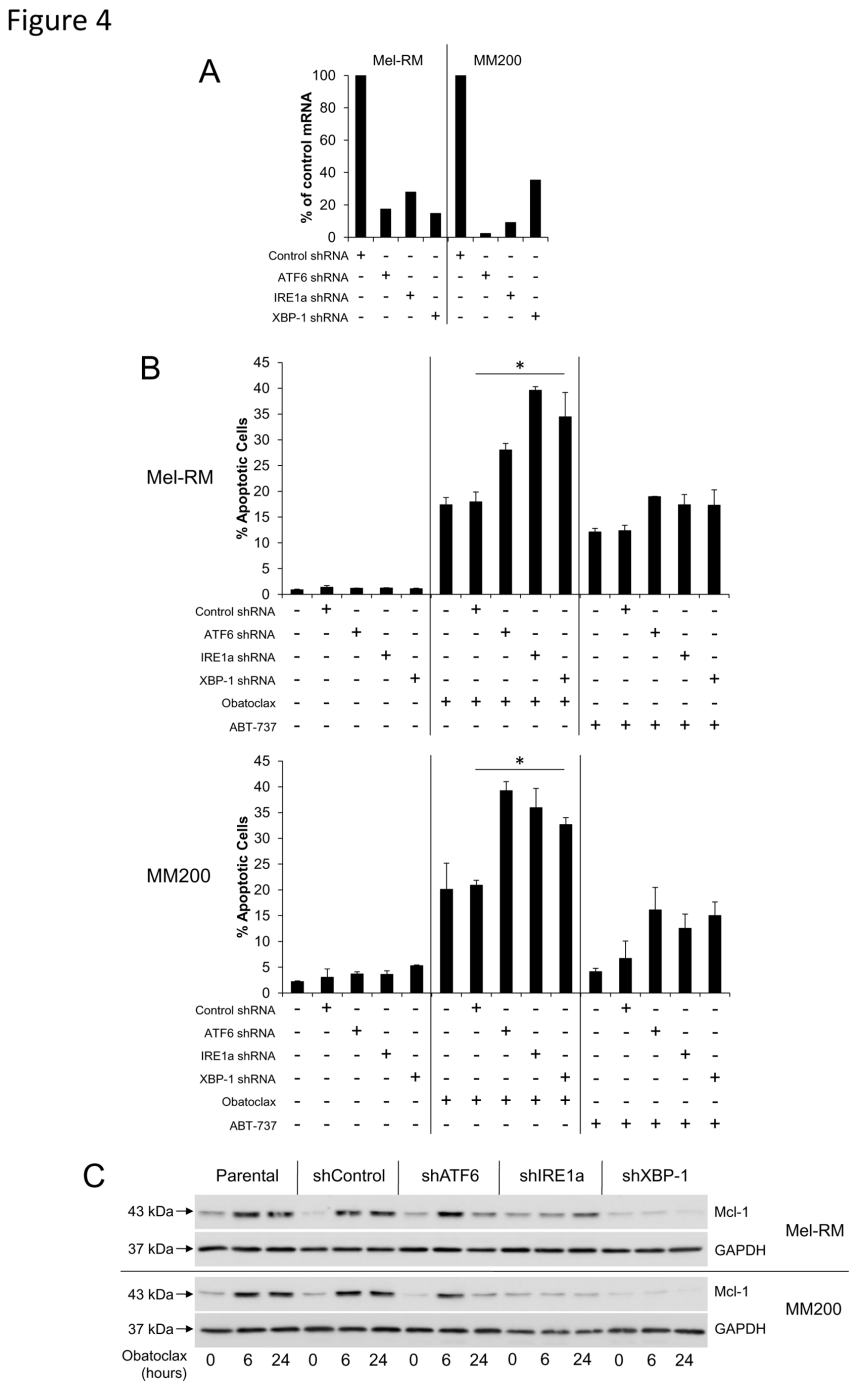
Inhibiting the UPR sensitises melanoma cells to Obatoclax- and ABT-737-induced cell death. A. Stable knockdowns of ATF6, IRE1α and XBP-1 were generated in Mel-RM and MM200 cells by retroviral insertion of shRNA. Transduced cells were selected with 5µg/ml puromycin. Knockdown of ATF6, IRE1α and XBP-1 was confirmed by real time-PCR. B. Following treatment of knockdown cells with Obatoclax (1µM) or ABT-737 (10µM) for 24 hours, measurement of apoptosis was performed by the propidium iodide method using flow cytometry. *Columns*, mean of three individual experiments; bars, SE. C. The stable knockdown cells were treated with Obatoclax (1µM) or ABT-737 (10µM) for indicated periods. Whole cell lysate was then subjected to Western blot analysis of Mcl-1 protein expression levels. Data are representative of three individual experiments.

To establish a relationship between upregulation of Mcl-1 and induction of the UPR in response to treatment with Obatoclax and ABT-737, we used shRNA to knock down expression of four proteins involved in the UPR - ATF6, IRE1α and XBP-1- and determined the subsequent effect on sensitivity to each compound. Figure 4B shows that following 24 hours of treatment, sensitivity of melanoma cells to Obatoclax was increased, most evidently in the ATF6, IRE1α and XBP-1 knockdown cells. Sensitivity to ABT-737 was also increased, albeit to a lesser extent. Additionally, Figure 4C indicates that while Mcl-1 was upregulated in the untransfected and control shRNA-transfected cells following 6 and 24 hours of treatment with Obatoclax or ABT-737, we observed that this upregulation of Mcl-1 was inhibited in the knockdown cells, particularly in those lacking IRE1α and XBP-1. Furthermore, basal expression of Mcl-1 was noticeably lower in cells lacking XBP-1.

### Induction of the UPR by ABT-737 is associated with increases in cytosolic Ca^2+^

**Figure 5:**
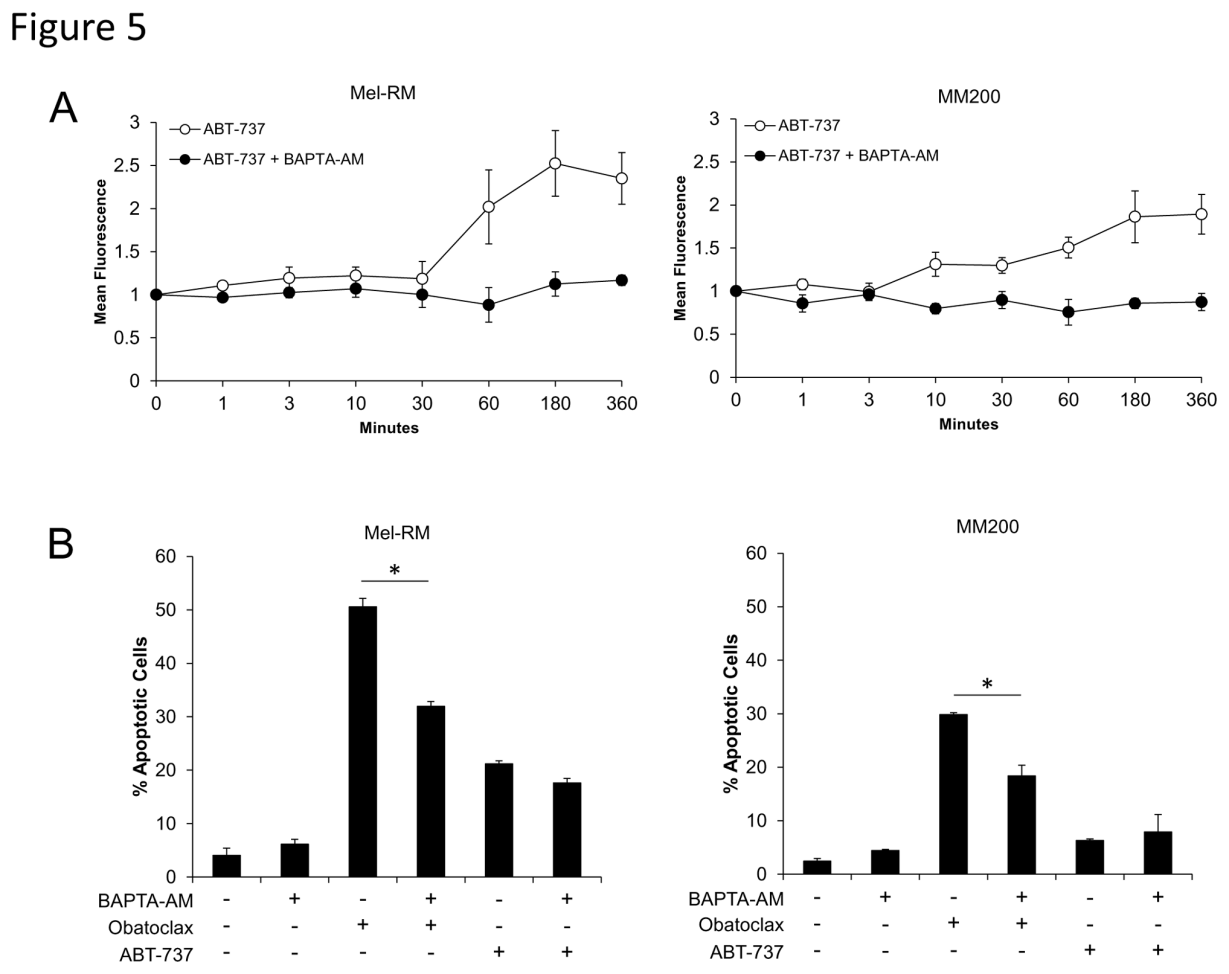
Induction of the UPR by ABT-737 is associated with increases in cytosolic Ca^2+^. A. Mel-RM and MM200 cells were treated with ABT-737 for indicated periods. Cells were collected, washed and incubated with 1µM of the fluorescent calcium probe Calcium Green-1 before analysis of calcium levels by flow cytometry. Obatoclax was unable to be used due to the inherent fluorescence of the compound. Increase in mean fluorescence is indicative of an increase in cytosolic calcium. Where indicated, cells were pretreated for 4 hours with 10µM of the calcium chelator BAPTA-AM before addition of ABT-737. *Points*, mean of three individual experiments; *bars*, SE. B. Mel-RM and MM200 cells were treated for 48 hours with Obatoclax (1µM) or ABT-737 (10µM) with or without a 4 hour pretreatnent with various doses of BAPTA-AM. Cells were then subjected to measurement of apoptosis by the propidium iodide method. *Columns*, mean of three individual experiments; *bars*, SE.

The ER is a site of intracellular calcium storage, with calcium homeostasis partially regulated by Bcl-2 family proteins located on the ER membrane [30]. Depletion of ER Ca^2+^ stores is also one event involved in the induction of ER stress. To investigate whether Obatoclax and ABT-737 induce ER stress through disruption of Ca^2+^ homeostasis by inhibition of Bcl-2 family proteins at the ER membrane, we measured changes in cytosolic Ca^2+^ levels following treatment with the agents. Treatment with ABT-737 resulted in increased cytosolic Ca^2+^ after 60 minutes in Mel-RM cells, peaking at 3-6 hours, and also to a lesser extent in MM200 cells (Figure 5A). Pretreatment of cells with a cell-permeant Ca^2+^ chelator, BAPTA-AM (10µM), was shown to inhibit the detection of increased cytosolic Ca^2+^ by ABT-737. Similar studies were not possible with Obatoclax due to interference from the inherent fluorescence of the compound with the Calcium Green-1 probe [21]. Nevertheless, as shown in Figure 5B, pretreatment with various doses of BAPTA-AM partially decreased the levels of apoptosis by Obatoclax after 48 hours, consistent with reduction in ER stress induced apoptosis resulting from chelation of cytosolic Ca^2+^. This was not observed following treatment with ABT-737, again consistent with the lower sensitivity to this agent compared to Obatoclax.

### Obatoclax, but not ABT-737, induces autophagy in human melanoma cells

Previous studies have suggested that inhibitors of antiapoptotic proteins may also induce autophagy [31]. To investigate the possible role of autophagy in the observed effects of the BH3 mimetics in melanoma cells, we examined the conversion of cytosolic microtubule-associated protein light chain 3 (LC3-I) to its autophagosome-bound form, LC3-II, and the expression of p62, classic indicators of autophagic activity.

**Figure 6:**
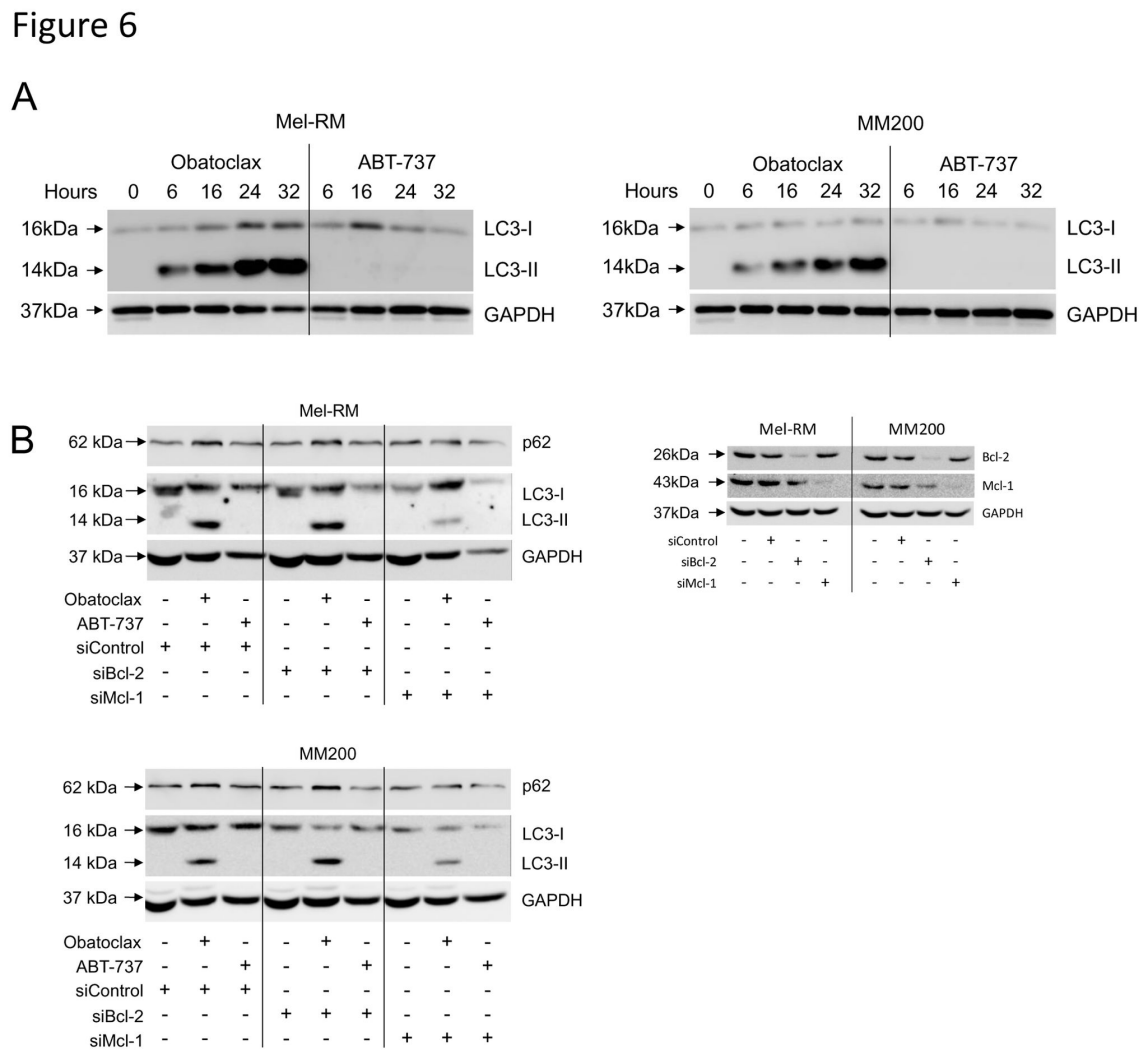
Induction of autophagy by BH3 mimetics. A. Mel-RM and MM200 cells were treated with Obatoclax (1µM) or ABT-737 (10µM) for indicated times. Whole cell lysates were collected and subjected to Western blot analysis for LC3 protein levels. The cytosolic form, LC3-I, is expressed as a 16kDa protein, which is lipidated and bound to autophagosome membranes, resolving as a 14kDa form, LC3-II. Data are representative of three individual experiments. B. Mel-RM and MM200 cells were transfected with either control, Bcl-2 or Mcl-1 siRNA before treatment with either Obatoclax (1µM) or ABT-737 (10µM) as indicated for 16 hours. Whole cell lysates were collected and subjected to Western blot analysis for Mcl-1 and Bcl-2, to confirm knockdown efficiency, and LC3 and p62 to investigate induction of autophagy. Data are representative of two individual experiments.

As shown in Figure 6A, there was a rapid increase in the lower molecular weight LC3-II following treatment with Obatoclax, indicating strong induction of autophagy. However, no LC3 conversion was detected in cells treated with ABT-737. This disparity was observed in a number of additional melanoma cell lines (data not shown). Accumulation of LC3-II was due to increased production of autophagosomes, as co-treatment with the late-stage autophagy inhibitor, Bafilomycin A1, led to a further increase in LC3-II levels (Figure S3). Levels of p62 decreased following an initial increase in Obatoclax-treated cells. Differences between the agents regarding induction of autophagy did not appear to be due to their respective binding affinities, in that siRNA knockdown of Mcl-1 did not induce autophagy in ABT-737 treated cells and had minimal effects on autophagy induced by Obatoclax (Figure 6B). We observed a small reduction in LC3-II levels in Obatoclax-treated Mcl-1 knockdown cells, which was not reversed in response to caspase inhibition (Figure S3). p62 expression was unaffected by knockdown of either protein. 

## Discussion

The above results indicate that treatment of melanoma cells with the BH3 mimetics Obatoclax and ABT-737 elicits a number of cellular responses related to inhibition of anti-apoptotic Bcl-2 family proteins, resulting in the induction of apoptosis. Obatoclax-induced apoptosis had relatively slow kinetics, with maximal apoptosis seen at 48 hours although changes in the morphology of melanoma cells were evident as early as 16 hours in some cell lines. In contrast, melanoma cells were relatively insensitive to treatment with ABT-737, with induction of cell death apparent only at doses exceeding 10µM. In addition, the morphological changes associated with exposure to Obatoclax were not observed in cells treated with ABT-737. This relative resistance to ABT-737 appeared largely due to Mcl-1 antiapoptotic proteins in the cell as RNAi silencing of Mcl-1 was sufficient to increase their sensitivity to ABT-737 induced apoptosis.

To further investigate the mechanism(s) of apoptosis induction we monitored changes in the pro- and anti-apoptotic proteins by Western blotting, using doses of Obatoclax which gave maximal apoptosis *in vitro* (1µM) as reported by other investigators [21,23,32-34]. The results showed that Obatoclax and ABT-737 induced marked increases in the pro-apoptotic Bcl-2 proteins Bim, Noxa and PUMA. Noxa appeared to play a role in Obatoclax-induced apoptosis in that siRNA knockdown resulted in substantial inhibition of apoptosis. Knockdown of Bim or PUMA, however, had no significant effect [32]. Unexpectedly, the current study showed that in addition to eliciting a pro-apoptotic response through induction of BH3-only proteins, both agents also induced a survival response by upregulation of the anti-apoptotic proteins Bcl-2, Bcl-XL and Mcl-1 that appeared to be due to induction of ER stress. This was supported by upregulation of known markers of ER stress such as GRP78, phosphorylation (and activation) of the eIF2α transcription factor and splicing of XBP-1. Inhibition of ER stress responses by siRNA knockdown of three proteins involved in the UPR - ATF6, IRE1α and XBP-1 - confirmed the role of ER stress in the Obatoclax- and ABT-737-induced upregulation of Mcl-1, resulting in increased sensitivity to the agents. Along with the observation that Mcl-1 is transcriptionally upregulated by Obatoclax, these results are consistent with previous studies from this laboratory [7], which have shown that Bcl-2 and Mcl-1 are trancriptionally upregulated during ER stress and that Mcl-1 is particularly important in protection of melanoma cells against apoptosis. Noxa and PUMA were similarly upregulated by transcription during ER stress and were shown to mediate apoptosis when Mcl-1 was knocked down [7]. Further studies have demonstrated that the IRE1α/XBP-1 arm of the UPR is key to regulating Mcl-1 expression during ER stress, through activation of the transcription factor Ets-1 [35]. Since ATF6 does not directly activate XBP-1, instead regulating its expression, it is likely to play a comparatively smaller role in Mcl-1 expression. Consistent with these reports, the current study demonstrates nearly complete suppression of Mcl-1 upregulation in cells lacking IRE1α and XBP-1.

The mechanism by which the two BH3 mimetics induce ER stress remains under investigation but may involve inhibition of the protective effects of the anti-apoptotic proteins on the ER [36]. Induction of ER stress is partially regulated by the control of intracellular calcium homeostasis by Bcl-2 located on the ER membrane [30]. Inhibition of Bcl-2 at the ER membrane can therefore lead to disturbances in calcium flux and subsequent induction of ER stress. The potential for a BH3 mimetic to induce ER stress is supported by the observation that the cellular distribution of Obatoclax is dependent on sites of Bcl-2 expression [21]. Unfortunately, investigation of calcium flux in response to Obatoclax was not possible due to the inherent fluorescence of the compound [21]; however, an increase in cytosolic Ca^2+^ was detected following treatment with ABT-737. This rapid increase in Ca^2+^ appeared to correlate with the kinetics of ER stress induced by the agent, which was initiated as early as 6 hours post treatment. Furthermore, this early event was shown to have a partial role in the induction of apoptosis as shown by reduction in apoptosis when the cells were treated with the Ca^2+^ chelator BAPTA-AM.

Additionally, this study highlights the ability of Obatoclax, but not ABT-737, to induce a strong and rapid autophagic response in melanoma cells. Characteristic changes in cell morphology, along with early and sustained upregulation of the autophagosome-associated LC3-II, occurred following treatment with Obatoclax alone. Knockdown of Mcl-1 was insufficient to facilitate ABT-737-induced autophagy, however we observed some decrease in LC3-II levels following treatment with Obatoclax. As the decrease could not be reversed by caspase inhibition, these observations support a role for Mcl-1 in the regulation of autophagy as previously described [37], through association of Mcl-1 with Beclin-1. We also observed a decrease in p62 expression following an initial accumulation in Obatoclax-treated cells, implying a defect in autophagosome degradation as reported previously [38]. This was shown to be a result of Obatoclax concurrently downregulating cathepsin expression [39], restricting completion of autophagy and consequently the potential for recovery, contributing to cell death.

These studies suggest that Obatoclax may be more effective than ABT-737 in treating melanoma because of its ability to target Mcl-1. In support of this, expression of Mcl-1 has been shown to be the major cause of resistance of melanoma cells to apoptosis induced by ABT-737, which inhibits all anti-apoptotic Bcl-2 family proteins except Mcl-1 and A1 [11-13]. Specifically, while the affinity of ABT-737 to Bcl-2 and Bcl-XL (IC_50_ of 0.12µM and 0.064µM respectively) is greater than that of Obatoclax (1.11µM and 4.69µM respectively), the IC_50_ of Obatoclax for Mcl-1 is 2.90µM, compared to >20µM for ABT-737 [40]. Additionally, our previous studies on tissue sections have shown that Mcl-1 and Bcl-XL expression is associated with progression of melanoma, whereas Bcl-2 levels decrease during progression of melanoma [41]. These findings highlight the necessity of inhibiting Mcl-1 to induce apoptosis in melanoma cells, and suggest that combinations of BH3 mimetics and inhibitors against other survival pathways may minimise anti-apoptotic effects, thus enhancing induction of apoptosis. A number of combinations are possible such as with MEK/ERK inhibitors and TRAIL, and results with such agents are the subject of further studies.

BH3 mimetics are traditionally described as agents which induce apoptosis primarily by binding to the anti-apoptotic proteins at the outer mitochondrial membrane, so freeing Bax and/or Bak with subsequent release of apoptogenic factors from the mitochondria [10,11]. To support this view, Obatoclax has been shown to interfere with interaction between Mcl-1 and Bak in SkMel-5 cells [21], in cholangiocarcinoma cells [42] and in acute myeloid leukaemia cells [23]. Further, Obatoclax could not induce apoptosis in mouse kidney epithelial cells lacking Bax and Bak [21]. We have also previously demonstrated partial inhibition of Obatoclax-induced apoptosis in Bak- or Bax-deficient melanoma cells [32]. However, it has also been reported that while ABT-737 is almost entirely dependent on activation of Bax/Bak and caspase 9, Obatoclax was reported to induce apoptosis in MEFs lacking both Bax and Bak [43]. In the present studies we found that Obatoclax was a strong inducer of autophagy which, along with the observation of caspase-independent cell death raises a question as to whether this was a more important inducer of apoptosis than activation of caspases as reported in studies on rhabdomyosarcoma cells [34] and breast cancer cells [38]. Further studies are needed to clarify this question in melanoma cells, but it seems possible that Obatoclax may induce cell type-specific mechanisms in addition to or independent of Bax/Bak mediated apoptosis. These supposed off-target effects may be a consequence of the widespread localisation of target Bcl-2 family proteins, for example at the outer nuclear envelope, and lysosomal and ER membranes.

In summary, the present studies show that both BH3 mimetics induce strong ER stress responses in melanoma cells, which limit the induction of apoptosis due principally to ER stress-induced upregulation of the anti-apoptotic protein Mcl-1. The increased sensitivity of melanoma to Obatoclax is consistent with the ability of Obatoclax, but not ABT-737, to inhibit Mcl-1. Obatoclax, but not ABT-737, was also found to be a strong inducer of autophagy, suggesting Mcl-1 plays a regulatory role in autophagy and which may have contributed to the increased sensitivity of melanoma to Obatoclax. These results support the conduct of further studies on the effectiveness of Obatoclax in combination with other agents that have activity against melanoma.

## Supporting Information

Figure S1 **Obatoclax-induced cell death is partially caspase-dependent.** A. Mel-RM and MM200 cells were pretreated as indicated for one hour with 30µM of the pan-caspase inhibitor, z-VAD-fmk, before treatment with 200ng/mL TRAIL or 1µM Obatoclax for 48 hours. Cell death was then measured by the propidium iodide method using flow cytometry. *Columns*, mean of three individual experiments; *bars*, SE.
(TIF)

Figure S2 **Obatoclax transcriptionally upregulates Mcl-1 in melanoma cells.** A. Mel-RM and MM200 cells were treated with 1µM Obatoclax for indicated timepoints. Cells were then harvested and Mcl-1 mRNA determined by RT-PCR. *Columns*, mean of three individual experiments; *bars*, SE.
(TIF)

Figure S3 **Induction of autophagy by Obatoclax.** A. Mel-RM and MM200 cells were pre-treated for one hour with 100nM Bafilomycin A1 where indicated, before treatment with 1µM Obatoclax for 16 hours. Whole cell lysate was collected and subjected to Western blot analysis for LC3. As Bafilomycin A1 inhibits formation of the autophagolysosome, resulting in accumulation of LC3-II, co-treatment with an autophagy-inducing agent further increases expression of LC3-II as autophagosomes are created and not degraded.
B. Mel-RM and MM200 cells were treated with 1µM Obatoclax for indicated timepoints, before collection of whole cell lysate for Western blot analysis of p62.
C. Mel-RM and MM200 cells were transfected with Mcl-1 siRNA as in Figure 6B, before treatment with 1µM Obatoclax, with or without z-VAD-fmk pretreatment (30µM, one hour). Whole cell lysate was collected and subjected to Western blot analysis for LC3 and p62.
(TIF)
