# Supplementary figures and images for: OBATOCLAX and ABT-737 Induce ER Stress Responses in Human Melanoma Cells that Limit Induction of Apoptosis
Source: PLoS One. 2013 Dec 19;8(12):e84073. doi: 10.1371/journal.pone.0084073 (PMC3868604; doi:10.1371/journal.pone.0084073)

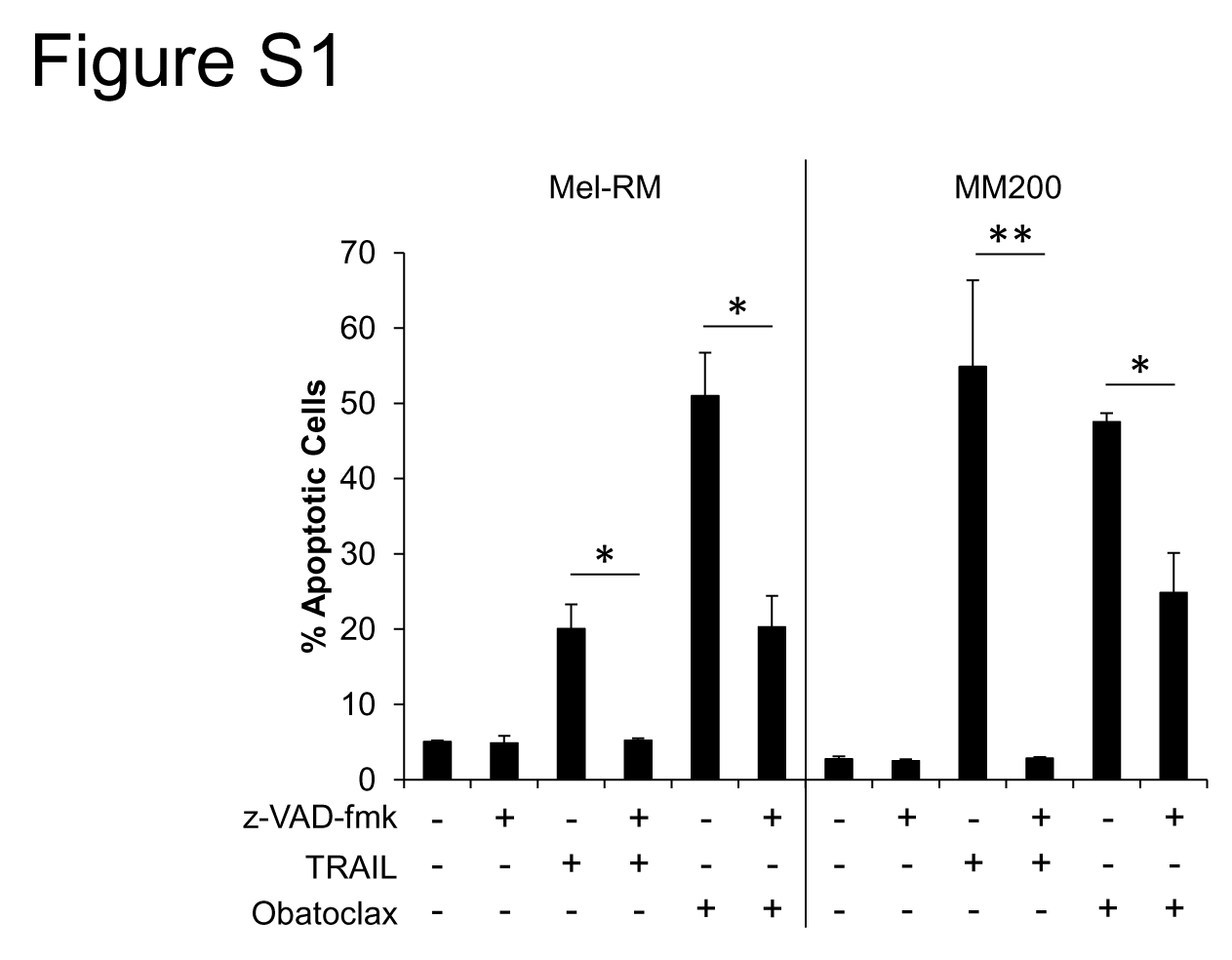

Supplement: Figure S1 — Obatoclax-induced cell death is partially caspase-dependent. A. Mel-RM and MM200 cells were pretreated as indicated for one hour with 30µM of the pan-caspase inhibitor, z-VAD-fmk, before treatment with 200ng/mL TRAIL or 1µM Obatoclax for 48 hours. Cell death was then measured by the propidium iodide method using flow cytometry. Columns, mean of three individual experiments; bars, SE. (TIF) [file pone.0084073.s001.tif]

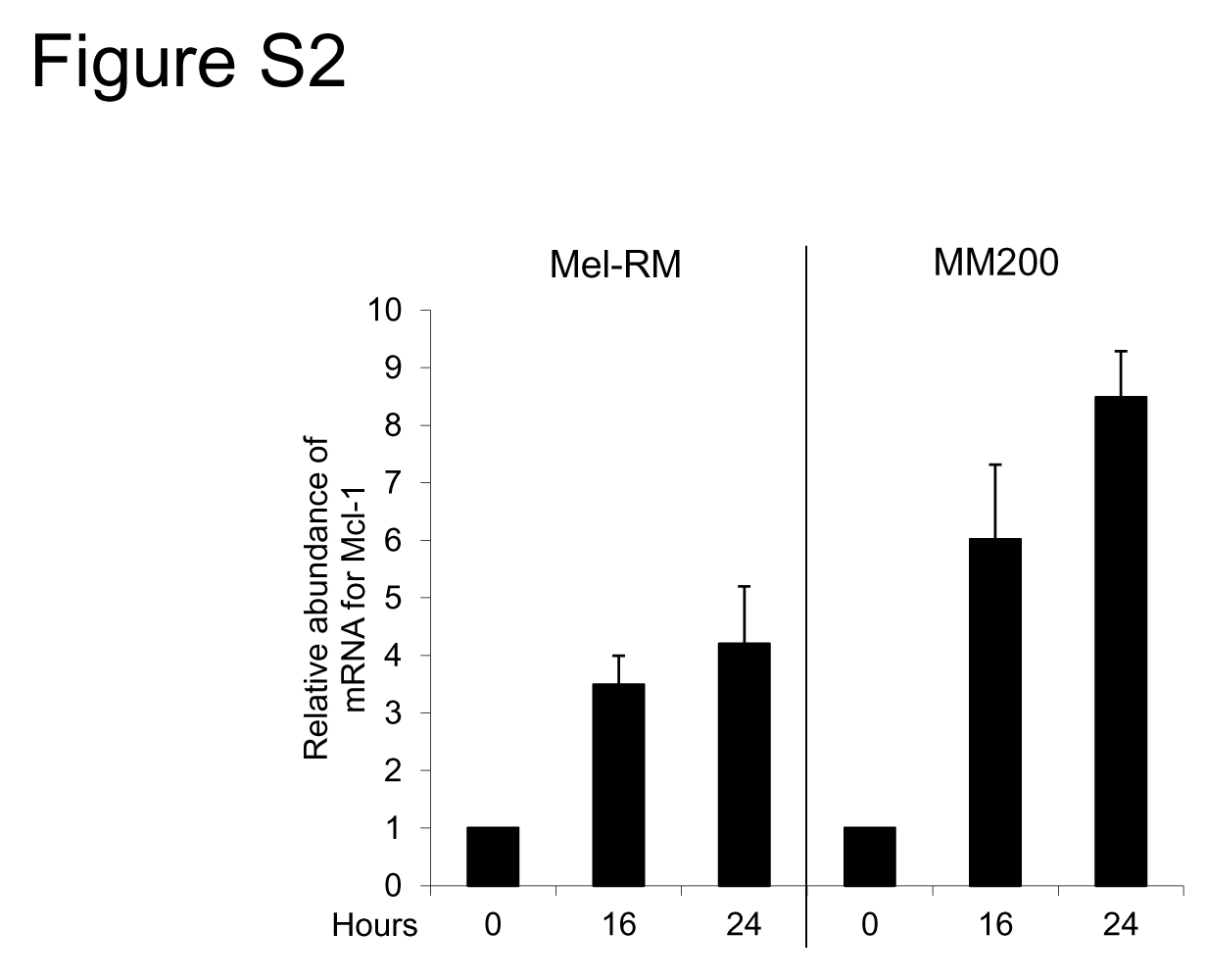

Supplement: Figure S2 — Obatoclax transcriptionally upregulates Mcl-1 in melanoma cells. A. Mel-RM and MM200 cells were treated with 1µM Obatoclax for indicated timepoints. Cells were then harvested and Mcl-1 mRNA determined by RT-PCR. Columns, mean of three individual experiments; bars, SE. (TIF) [file pone.0084073.s002.tif]

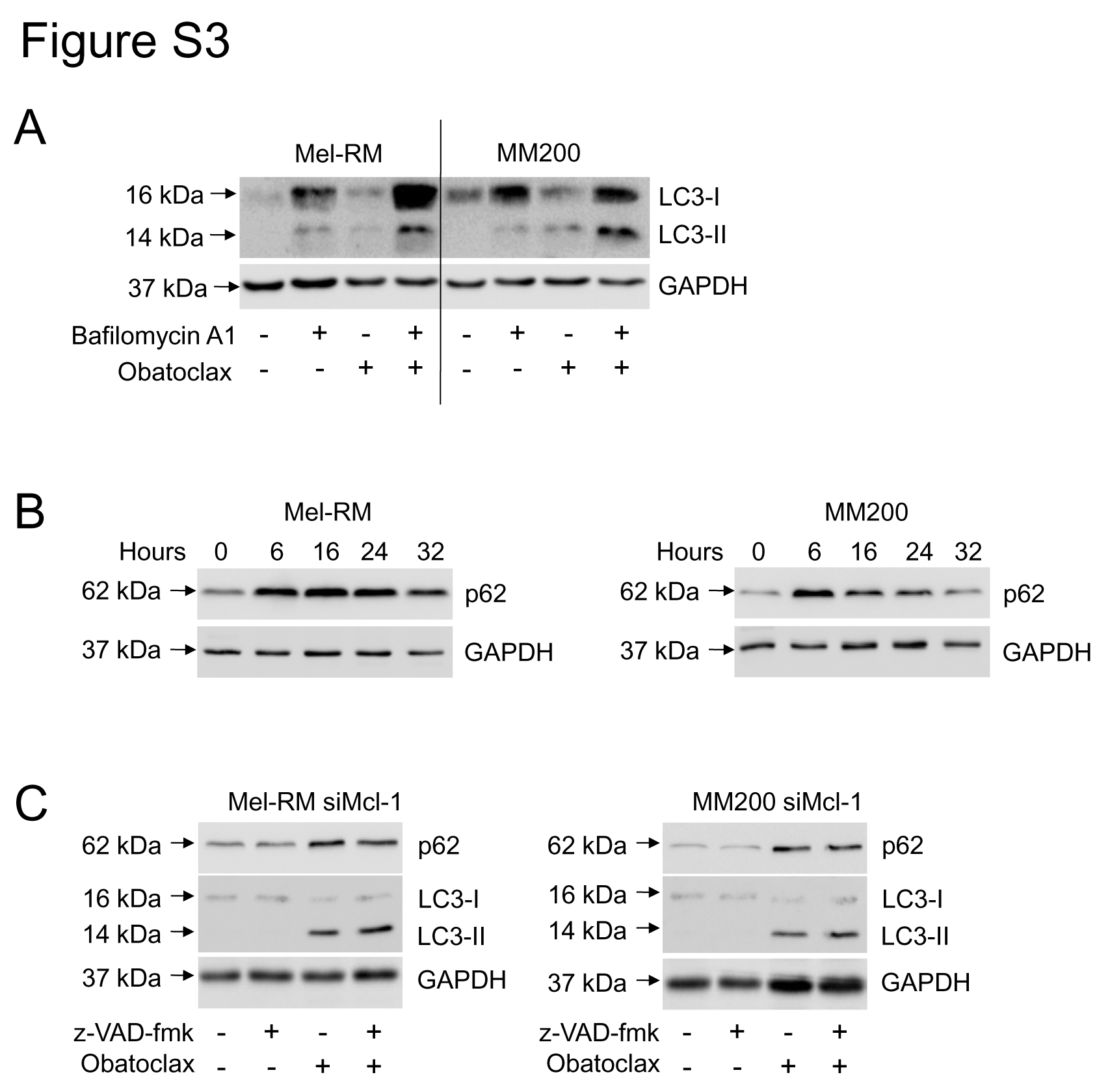

Supplement: Figure S3 — Induction of autophagy by Obatoclax. A. Mel-RM and MM200 cells were pre-treated for one hour with 100nM Bafilomycin A1 where indicated, before treatment with 1µM Obatoclax for 16 hours. Whole cell lysate was collected and subjected to Western blot analysis for LC3. As Bafilomycin A1 inhibits formation of the autophagolysosome, resulting in accumulation of LC3-II, co-treatment with an autophagy-inducing agent further increases expression of LC3-II as autophagosomes are created and not degraded. B. Mel-RM and MM200 cells were treated with 1µM Obatoclax for indicated timepoints, before collection of whole cell lysate for Western blot analysis of p62. C. Mel-RM and MM200 cells were transfected with Mcl-1 siRNA as in Figure 6B, before treatment with 1µM Obatoclax, with or without z-VAD-fmk pretreatment (30µM, one hour). Whole cell lysate was collected and subjected to Western blot analysis for LC3 and p62. (TIF) [file pone.0084073.s003.tif]
